# Amyopathic Dermatomyositis Associated with Histopathological Findings of Organizing Pneumonia and Pulmonary Vasculitis

**DOI:** 10.4274/balkanmedj.2016.1061

**Published:** 2017-08-04

**Authors:** Jeong Uk Lim, Hye Seon Kang, Yong Hyun Kim, Tae-Jung Kim

**Affiliations:** 1 Division of Allergy and Pulmonology, Department of Internal Medicine, Bucheon St. Mary’s Hospital, The Catholic University of Korea School of Medicine, Seoul, South Korea; 2 Department of Hospital Pathology, The Catholic University of Korea School of Medicine, Seoul, South Korea

**Keywords:** Amyopathic dermatomyositis, interstitial lung disease, organising pneumonia, pulmonary vasculitis

## Abstract

**Background:**

Clinically, amyopathic dermatomyositis is a clinically distinct subgroup of dermatomyositis characterised by unique dermatological manifestations without muscle involvement. Clinically, amyopathic dermatomyositis is frequently associated with interstitial lung disease, which usually has a rapidly progressive, fatal clinical course. Although clinically, amyopathic dermatomyositis-related interstitial lung disease is well described, data on the histopathology of clinically, amyopathic dermatomyositis-interstitial lung disease are limited. Organising pneumonia and pulmonary vasculitis have rarely been reported.

**Case Report:**

A 54-year-old Korean woman presented with exertional dyspnoea and a dry cough. Chest computed tomography revealed subpleural ground-glass opacities suggesting interstitial lung disease, which was later pathologically confirmed to be a combination of organising pneumonia and pulmonary vasculitis. The patient improved markedly with prednisone treatment and has remained stable for a long time.

**Conclusion:**

We hereby report a rare combination of organising pneumonia and pulmonary vasculitis in a patient with amyopathic dermatomyositis-interstitial lung disease.

Clinically, amyopathic dermatomyositis (CADM) is a variant phenotype of dermatomyositis defined by characteristic skin manifestations such as a heliotrope rash and Gottron’s papules (1), but little or no evidence of myopathy_ENREF_2. Arthralgia, fever, and Raynaud’s phenomenon are other clinical manifestations of CADM (1). Although the absolute number of CADM cases is small compared with other inflammatory myopathies, the prevalence of interstitial lung disease (ILD), a serious extramuscular manifestation of CADM and a major complication of dermatomyositis and polymyositis, is quite significant (2). The prevalence of ILD in patients with CADM is higher in Korea and Japan than in Western countries (1)_ENREF_1. However, there are very few data on the histopathology of CADM-ILD, which may vary among patients (3). No treatment for ILD with CADM has been established because no large studies have been performed. We believe that the pathological type of ILD is a significant factor related to the outcome of CADM, because the response to conventional therapy varies according to the ILD subtype. 

We herein present a rare case of CADM-ILD with organising pneumonia and pulmonary vasculitis that showed a rapid, prolonged response to steroid treatment.

## CASE PRESENTATION

**Figure 1:**
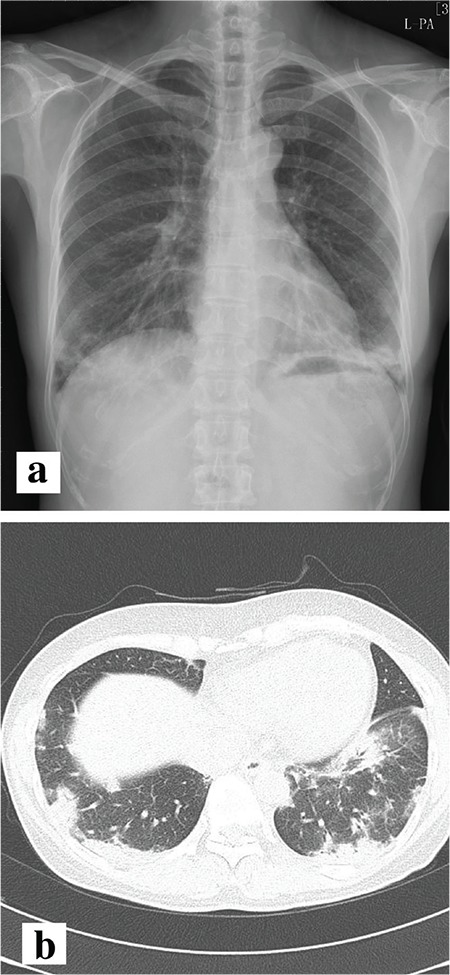
The initial chest X-ray showed pneumonic consolidations in both lower lung fields (a). Chest computed tomography showed patchy subpleural ground-glass opacities and pneumonic consolidations in the lower lobes of both lungs (b).

**Figure 2:**
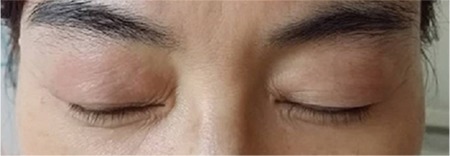
Swelling and redness of both upper eyelids, consistent with a heliotrope rash.

**Figure 3:**
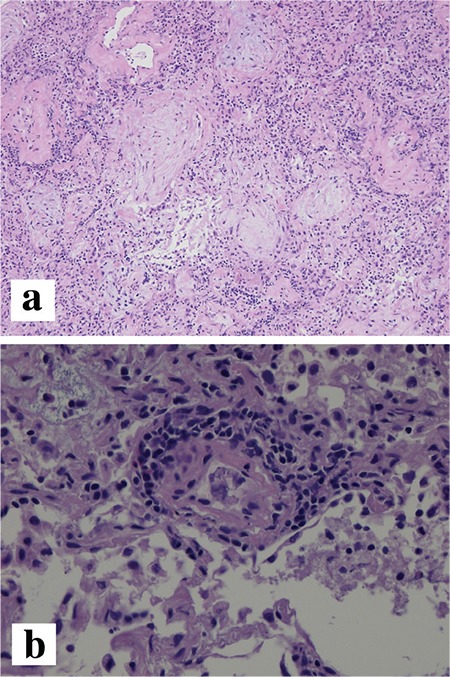
A biopsy obtained via video-assisted thoracoscopic surgery shows loose organising fibrinoid tissues within the alveolar spaces (H&E, x200) (a). The same specimen shows neutrophilic infiltration around the pulmonary vessel walls consistent with pulmonary vasculitis (H&E, x400) (b).

A 54-year-old woman presented with a three-week history of exertional dyspnoea and a dry cough. Fine crackles were heard at the base of both lungs. The patient had no fever, arthralgia, or myalgia, and she did not complain of any symptoms suggestive of muscle weakness. Her medical history was unremarkable. She had no history of smoking or heavy alcohol consumption. Her family history was also unremarkable. An initial chest X-ray showed consolidations in both lung fields (Figure 1a). Chest computed tomography showed ground-glass opacities and subpleural curvilinear shadows in the lower lobes of both lungs (Figure 1b). The patient had periorbital erythema of both eyes, which appeared after the initiation of the respiratory symptoms, consistent with a heliotrope rash (Figure 2). In pulmonary function tests, the vital capacity was mildly decreased to 2.40 L (75% of the predicted value), and the carbon monoxide diffusing capacity was decreased to 10.2 mL/mmHg/min (53% of the predicted value). The creatinine phosphokinase level was 81 U/L, within the normal range, and the lactate dehydrogenase level was mildly elevated at 625 U/L. Anti-cyclic citrullinated protein (CCP) antibody (109.8 U/mL) was positive, and antinuclear antibody was negative. Rheumatoid factor and anti-DNA, anti-glomerular basement membrane, anti-Jo-1, and anti-Ro/La antibodies were also negative. Diagnostic fibre optic bronchoscopy was performed. Bronchoalveolar lavage fluid analysis revealed 3% neutrophils, 36% lymphocytes, 12% eosinophils, and 49% macrophages. No microbes were identified from bronchoalveolar lavage fluid culture. For histological confirmation, wedge resection was performed via video-assisted thoracoscopic surgery. The pathological findings were consistent with organising pneumonia (Figure 3) and pulmonary alveolar vasculitis. Considering the preliminary diagnosis of CADM-ILD, prednisolone was administered at 0.5 mg/kg body weight per day. The patient’s symptoms improved dramatically, and the skin lesions around her eyes also disappeared within two months. A follow-up X-ray showed regression of the initial pulmonary infiltration. The prednisolone was subsequently tapered to a maintenance dose. During the subsequent two years, the patient developed no clinical manifestations such as muscle weakness or arthralgia/arthritis.

Written informed consent was obtained directly from the patient presented in this report.

## DISCUSSION

A significant association between CADM and ILD has been described. The prevalence of ILD is higher in patients with CADM than in those with classic dermatomyositis or polymyositis. The reported prevalence of ILD in Korean patients with CADM is 83.3%, _ENREF_6 while Yamanishi reported that 71% of 21 Japanese patients with ADM had ILD (4). In Asian populations, CADM-ILD is reportedly rapidly progressive and frequently unresponsive to glucocorticoid treatment, leading to poor survival (3)_ENREF_8. There are few reports on the pathological features of CADM-ILD, and the relationship between the clinical outcome and pathological diagnosis is not clear. The histological patterns of CADM-ILD vary and include desquamative interstitial pneumonitis, usual interstitial pneumonia (UIP), non-specific interstitial pneumonia (NSIP), and diffuse alveolar damage (DAD) (5). Kang et al. (4) described lung biopsy results in their cohort of inflammatory myopathy, including six patients with CADM. They found that UIP, DAD, UIP with DAD, and NSIP patterns predominated, but they did not clarify the pathological pattern of CADM specifically. Suda et al. (3) reported that the most common pathological type was NSIP (five of eight patients with CADM). A recent larger study found 12 cases of NSIP, two cases of UIP, and five cases of DAD among 19 patients with CADM-ILD who underwent surgical lung biopsies (6). Overall, NSIP might be the most common histopathological pattern of CADM-ILD. According to previous studies on ILD, organising pneumonia is rare in patients with CADM-ILD (7). 

Confirmation of the pathological types of CADM-ILD should help to guide treatment, because different pathological types may show variable responses to steroid therapy. A case of pulmonary vasculitis in a patient with dermatomyositis has been reported (6). Only one out of 197 CADM patients had organising pneumonia according to the review by Gerami et al. (7) and pulmonary vasculitis has not been reported in CADM-ILD patients. Considering that CADM in itself is a subtype of dermatomyositis, simultaneous discovery of the two pathological findings in one patient is extremely rare. For our patient, after initiating steroid therapy the respiratory symptoms and skin lesion subsided in four weeks and the lung consolidations had disappeared in a follow-up chest X-ray. As mentioned above, CADM-ILD is usually fatal. The unique pathological type is a possible contributing factor to the favourable response to treatment (8).

Our case was also unique in that the positive anti-CCP antibody titre at both the time of diagnosis and during the follow-up suggests the possibility of rheumatoid arthritis (RA). The clinical significance of the positive anti-CCP antibody titre without apparent manifestations of RA is not clear, but it has been proposed that patients with ILD and the presence of anti-CCP antibodies are at risk of developing RA (9). Alison suggested that the lung is the site of the initial RA-related immune dysregulation and that interactions between genetic and environmental factors lead to localised lung injury and contribute to the subsequent development of ILD (10). 

In conclusion, we have reported a case of CADM-ILD that was pathologically confirmed to be a combination of organising pneumonia and pulmonary vasculitis. The patient was persistently positive for anti-CCP antibody without apparent signs of RA and was successfully treated with steroids.
